# The Effect of Broccoli Glucosinolates Hydrolysis Products on *Botrytis cinerea*: A Potential New Antifungal Agent

**DOI:** 10.3390/ijms25147945

**Published:** 2024-07-20

**Authors:** Juan Román, Ailine Lagos, Andrea Mahn, Julián Quintero

**Affiliations:** Department of Chemical Engineering, University of Santiago, Chile, Avenida Libertador Bernardo O’Higgins 3363, Estación Central, Santiago 9170019, Chile; ailine.lagos@usach.cl (A.L.); andrea.mahn@usach.cl (A.M.)

**Keywords:** broccoli, glucosinolates hydrolysis products, *Botrytis cinerea*, antifungal activity, agricultural applications

## Abstract

The present study investigates the interactions between eight glucosinolate hydrolysis products (GHPs) sourced from broccoli by-products and the detoxifying enzymes of *Botrytis cinerea*, namely eburicol 14-alpha-demethylase (CYP51) and glutathione-S-transferase (GST), through in silico analysis. Additionally, in vitro assays were conducted to explore the impact of these compounds on fungal growth. Our findings reveal that GHPs exhibit greater efficacy in inhibiting conidia germination compared to mycelium growth. Furthermore, the results demonstrate the antifungal activity of glucosinolate hydrolysis products derived from various parts of the broccoli plant, including inflorescences, leaves, and stems, against *B. cinerea*. Importantly, the results suggest that these hydrolysis products interact with the detoxifying enzymes of the fungus, potentially contributing to their antifungal properties. Extracts rich in GHPs, particularly iberin and indole-GHPs, derived from broccoli by-products emerge as promising candidates for biofungicidal applications, offering a sustainable and novel approach to plant protection by harnessing bioactive compounds from agricultural residues.

## 1. Introduction

Plant diseases caused by fungi present significant challenges in agriculture, leading to considerable losses in crop yield and quality [1,2,3]. Among these pathogens, *Botrytis cinerea* is a common fungal phytopathogen that can damage a wide variety of crops due to its ability to cause the “gray mold” condition, resulting in detrimental crop consequences [4]. The impact of this fungal phytopathogen is significant, causing substantial economic losses in agricultural systems due to reduced crop yield and compromised quality [2,5]. Given the destructive nature of *B. cinerea*, the necessity for effective antifungal strategies in crop protection is very important to safeguard crop productivity and ensure food security [6].

In response to these challenges, plant-derived natural compounds are widely recognized for their diverse biological activities, including antifungal properties [7,8].

Glucosinolates (GLS) are sulfur and nitrogen-containing secondary metabolites commonly found in the *Brassicaceae* family [9]. Their structure includes a molecule of α-D-glucopyranose, linked to a hydroxylamine sulfate ester via a sulfur bridge, along with a variable side chain derived from aliphatic (leucine, isoleucine, methionine, or valine), aromatic (tyrosine or phenylalanine), or indolic (tryptophan) amino acids [10,11,12]. Over 200 types of GLS have been identified, and within a single species, more than 15 different types have been reported [13,14]. These secondary metabolites are part of the plant’s defense mechanism against biotic and abiotic stress [15].

GLS, while initially non-bioactive, interacts with the enzyme β-thioglucosidase glucohydrolase (E.C. 3.2.1.147), also known as myrosinase, upon plant tissue disruption. This enzyme catalyzes the hydrolysis of the β-thioglucoside bond in GLS. When plant tissue is damaged, this process leads to the formation of glucosinolate hydrolysis products (GHPs), a range of biologically active compounds [16,17]. These include isothiocyanates, thiocyanates, nitriles, oxazolidine-2-thiones, and epithionitriles, which have demonstrated efficacy against a range of pathogens and phytopathogens, including bacterial strains like *Escherichia coli* and *Staphylococcus aureus*, as well as fungal strains such as *Sclerotinia sclerotiorum* and *Botrytis cinerea* [4,18,19,20].

Incorporating phytochemical compounds as synergistic agents with biocides has emerged as an effective method to combat multi-drug resistance in pathogens. Various plant extracts, essential oils, and pure isolated compounds have shown potential in inhibiting enzymes that contribute to resistance [21]. Glucosinolates (GLS), found in *Brassicaceae*, including broccoli, are noteworthy due to their reactive hydrolysis products formed by the enzyme myrosinase upon plant tissue disruption. These hydrolysis products include isothiocyanates, nitriles, and other compounds with notable antifungal properties [22,23].

The investigation of CYP51 and GST is strategically significant due to their crucial roles in the detoxification mechanisms of *B. cinerea*. CYP51, also known as 14-alpha-demethylase (EC 1.14.14.154), is involved in the biosynthesis of ergosterol, a vital component of fungal cell membranes. Inhibiting this enzyme disrupts ergosterol production, compromising cell membrane integrity and leading to fungal cell death. This enzyme has been reported as a cause of resistance to several fungicides, including fenhexamid, pyrisoxazole, and tebuconazole [24,25,26]. Similarly, GST, also known as glutathione-S-transferase (EC 2.5.1.18) is integral to the detoxification of reactive oxygen species and xenobiotics through conjugation with glutathione. This enzyme plays a role in the resistance to fungicides such as captan, chlorothalonil, and dicarboximides (Leroux et al., 2002). Targeting these enzymes with glucosinolate hydrolysis products (GHPs) can effectively impair the fungus’s detoxification mechanisms and enhance the antifungal efficacy.

A few studies have demonstrated the significant antifungal activity of ITCs, particularly against *Botrytis cinerea* on strawberries [6]. Furthermore, a recent review highlights various antimicrobial agents as alternatives to synthetic fungicides, which include essential oils known for their effectiveness against numerous fungal pathogens, as well as plant extracts specifically used for the management of fungal rots in apples. The review also covers a range of studies on natural compounds such as methyl jasmonate, gibberellins, kinetins, auxins, and flavonoids, all demonstrating promising antifungal properties, thereby highlighting the broad potential of natural substances in agricultural disease management [3]. These studies highlight the remarkable inhibitory effects of plant-derived natural compounds against various fungal species. For this reason, the implementation of effective antifungal strategies utilizing GHPs can significantly contribute to disease management and mitigate the detrimental impact of phytopathogens on several susceptible crops.

Cruciferous vegetables, particularly broccoli, are rich in glucosinolates [27,28,29]. However, the edible parts of broccoli, corresponding to the inflorescences or florets, account for only 20–30% of the total biomass of the plant, while the remaining percentage consists of plant by-products, such as leaves and stems [30]. Broccoli is one of the vegetables that generates the most agro-industrial waste [31]. These by-products are often underutilized, despite their potential for antifungal applications [32].

This work aims to investigate the antifungal activity of hydrolysis products derived from glucosinolates found in broccoli inflorescences, leaves, and stems against *B. cinerea*. To achieve this goal, the GHPs were first identified using GC-MS/MS. Subsequently, molecular-docking simulations were performed to investigate the interactions between GHPs and the fungus’s detoxifying enzymes (CYP51 and GST). Additionally, antifungal activity assays were conducted using different concentrations of GHPs to evaluate the growth of mycelium and conidial germination of *B. cinerea*. This research significantly contributes to the development of effective antifungal strategies by demonstrating the potential application of GHPs as bioactive compounds. The findings of this study enhance our understanding of the antifungal properties of these compounds, providing a solid basis for the development of new and sustainable antifungal agents derived from broccoli glucosinolates. The results hold promise for future advancements in innovative and environmentally friendly solutions in the field of antifungal research.

## 2. Results

### 2.1. Identification of Glucosinolate Hydrolysis Products in Broccoli Inflorescences, Leaves, and Stems Using GC-MS

In this study, Gas Chromatography-Mass Spectrometry (GC-MS) analysis of broccoli revealed a unique distribution of glucosinolate hydrolysis products (GHPs) across its inflorescences, leaves, and stems. Eight GHPs were identified, including 1-butene, 4-isothiocyanato-indole, iberin nitrile, 5-methylindole, sulforaphane nitrile, iberin, 1H-indole-3-acetonitrile, and 1H-indole-3-carboxaldehyde. In the leaves, all these compounds were detected except for iberin nitrile. A comprehensive overview of each compound, including their retention time and peak area (% of total), is detailed in Table 1.

These compounds were categorized into aliphatic and indolic groups, with aliphatic GHPs including 1-butene, 4-isothiocyanate-indole, iberin nitrile, sulforaphane nitrile, and iberin, and indolic GHPs including indole, 5-methylindole, 1H-indole-3-acetonitrile, and 1H-indole-3-carboxaldehyde.

### 2.2. Structural Modeling and Refinement of B. cinerea CYP51 and GST Enzymes

To evaluate the interaction of the previously identified eight GHPs in broccoli, studies were conducted focusing on two *B. cinerea* enzymes, CYP51 and GST, which are reported to be key components of the pathogen’s defense mechanism against fungicides and other potentially toxic compounds.

In the current study, the three-dimensional structures of eburicol 14-alpha-demethylase (CYP51) and glutathione-S-transferase (GST) enzymes were modeled due to the absence of reported crystallographic structures to date. The structural three-dimensional models for each enzyme were built using the Iterative Threading ASSEmbly Refinement (I-TASSER) based on the reported sequence for CYP51 and GST. The CYP51 model showed a C-score of 1.04 and a TM-score of 0.86, indicative of a topology comprising 27 α-helices, 12 β-sheets, and 34 turns. The GST model had a C-score of −0.86 and a TM-score of 0.61, featuring 12 α-helices, 4 β-sheets, and 18 turns.

Additionally, in a comparative analysis of sequence identities, the Uniprot sequences utilized for CYP51 (Uniprot Code: Q9P428) and GST (Uniprot Code: Q9HF89) showed up to 68% and 48% identity, respectively, with the sequences employed by I-TASSER. 

**Figure 1:**
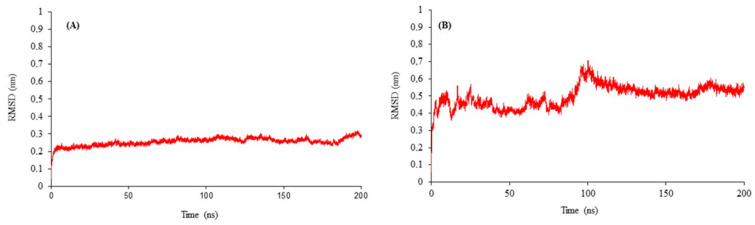
Molecular-Dynamics-Based Refinement of (**A**) CYP51 and (**B**) GST models over a 200 ns simulation. RMSD is the root mean square deviation.

To confirm the structural stability and dynamic behavior of the CYP51 and GST enzyme models, molecular dynamics simulations were carried out over 200 nanoseconds (ns). Figure 1 depicts the molecular dynamics refinement for the backbone atoms of (A) CYP51 and (B) GST models, focusing on the root mean square deviation (RMSD).

For CYP51, the RMSD values remained relatively stable during the simulation, oscillating within a narrow range of 0.2 to 0.3 nanometers (nm). On the other hand, the GST model exhibited more pronounced RMSD fluctuations, particularly within the initial 100 ns of simulation. This was followed by a period of relative stability extending up to 180 ns, with RMSD values ranging from 0.3 to 0.6 nm. 

The productive phase, defined as the period during which the enzyme maintains its optimal conformational state for catalytic activity, has been determined based on the RMSD data obtained from the MD simulations [33]. For CYP51, the productive phase is observed around 100 ns, where the RMSD values show minimal fluctuation and stabilize around 0.25 nm. In contrast, GST exhibits its productive phase around 150 ns, with RMSD values stabilizing at approximately 0.55 nm. These findings align with previous studies that have reported RMSD stability for complex biological macromolecules, such as enzymes with cofactors [23,34,35,36]. The convergence observed in our 200 ns simulations, ranging from 0.3 to 0.6 nm (3–6 Å), is consistent with the reported range for similar systems [23,37]. The Radius of Gyration (Rg) and Root Mean Square Fluctuation (RMSF) analyses for the GST enzyme were performed to further validate the structural stability observed in the MD simulations (Figure S1). The Rg plot indicates the compactness of the enzyme over the simulation period, while the RMSF plot highlights the flexibility of individual residues. These analyses are crucial in understanding the dynamic behavior of the enzyme and its interactions with various substrates.

**Figure 2:**
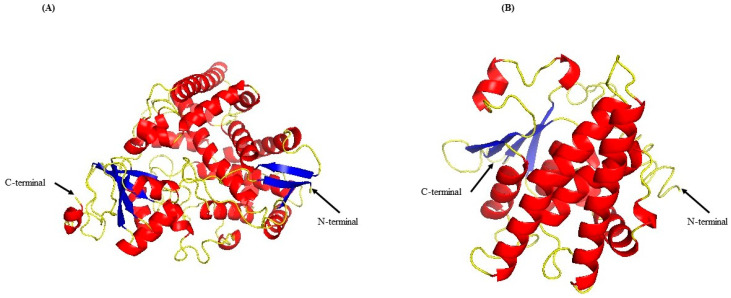
Refined Three-Dimensional Structure Models of B. cinerea Enzymes. The refined model of (**A**) CYP51 and (**B**) GST. Both models are depicted with the N-terminal and the C-terminal regions. The alpha helices are highlighted in red, the beta sheets in blue, and the coil regions in yellow. The models were deposited in the Protein Model Database under access codes PM0084614 for CYP51 and PM0084615 for GST, respectively.

The refined models of CYP51 and GST displayed slight differences in topology compared to the initial models proposed by I-TASSER. The CYP51 refined model (Figure 2A) displayed a topology with 32 α-helices, 10 β-sheets, and 40 turns. The GST model (Figure 2B) featured 12 α-helices, 3 β-sheets, and 22 turns. The 3D models of CYP51 and GST were further evaluated using QMEAN, SAVESv6.0 (ERRAT, VERIFY3D, PROCHECK), ProSA, and PDBsum. The results for CYP51 are as follows: a QMEANDisCo Global score of 0.77 ± 0.05; an ERRAT Overall Quality Factor of 94.2797; a VERIFY3D pass rate of 85.45%; PROCHECK showing 6 errors, 1 warning, and 1 pass; a ProSA Z-Score of −9.27; and a PDBsum Overall G-Factor of −0.33. For GST, the results are as follows: a QMEANDisCo Global score of 0.66 ± 0.05; an ERRAT Overall Quality Factor of 82.9268; a VERIFY3D pass rate of 91.34%; PROCHECK showing 7 errors and 2 warnings; a ProSA Z-Score of −4.08; and a PDBsum Overall G-Factor of −0.66. Despite these positive evaluations, molecular dynamics (MD) simulations were performed to relax the bonds and refine the structures of both models. Detailed figures are provided in the Supplementary Materials (Table S1). CYP51 and GST models were deposited in the Protein Model Data Base (PMDB) with the access codes PM0084614 and PM0084615, respectively (https://bioinformatics.cineca.it/PMDB/ accessed on 6 March 2023). These refined models were used for molecular-docking simulations.

### 2.3. Molecular Interactions of GHPs with B. cinerea CYP51 and GST Enzymes

Molecular-docking simulations were conducted to elucidate the interactions of Botrytis cinerea’s detoxifying enzymes, CYP51 and GST, with the previously identified glucosinolate hydrolysis products (GHPs). Table 2 shows the results of these docking simulations, detailing the affinity energy values (in kcal/mol) as determined using the Autodock Vina program. The table categorizes the data into substrates, inhibitors, and the eight specific GHPs previously determined, each evaluated for its interaction with the CYP51 and GST enzymes of Botrytis cinerea.

In this study, glutathione and obtusifoliol were utilized as reference substrates for identifying the binding sites on GST and CYP51 enzymes, respectively [38,39]. In the case of CYP51, the heme B group was included as a cofactor before ligand simulations, considering its essential role in the activity of CYP51 [40]. This approach facilitated the molecular-docking simulations. Additionally, these substrates served as baselines for comparing the affinity energies of diverse ligands, encompassing a range of inhibitors and GHPs. 

For inhibitory interaction reference, diniconazole, limonene, triadimefon, and vanillin were simulated. These molecules have been reported as inhibitors for enzymes in organisms that share similarities with the enzymes studied, such as Penicillium digitatum for CYP51 [41] and Phanerochaete chrysosporium for GST [40,42]. Additionally, interactions with carbendazim and tebuconazole, components of the commercially available ANASAC^®^ fungicide used for the control of various fungal diseases, as well as fluconazole, a broad-spectrum antifungal used in medicine, were simulated. 

In this study, fluconazole, along with carbendazim and tebuconazole, exhibited the most negative affinity energies. These findings indicate a robust inhibitory potential against the target enzymes. Fluconazole displayed notably negative affinity energies of −8.3 kcal/mol for CYP51 and −8.2 kcal/mol for GST, underscoring its strong inhibitory efficacy.

On the other hand, indolic GHPs, notably 3-Indoleacetonitrile, have exhibited substantial stability in their interactions with both CYP51 and GST enzymes of Botrytis cinerea. This stability is evidenced by an affinity energy of −6.4 kcal/mol, closely comparable to the affinity energy of glutathione (−6.7 kcal/mol). Similar stability profiles are observed with indolic GHPs like indole-3-carboxaldehyde, 6-methyl-1H-indole, and indole.

**Figure 3:**
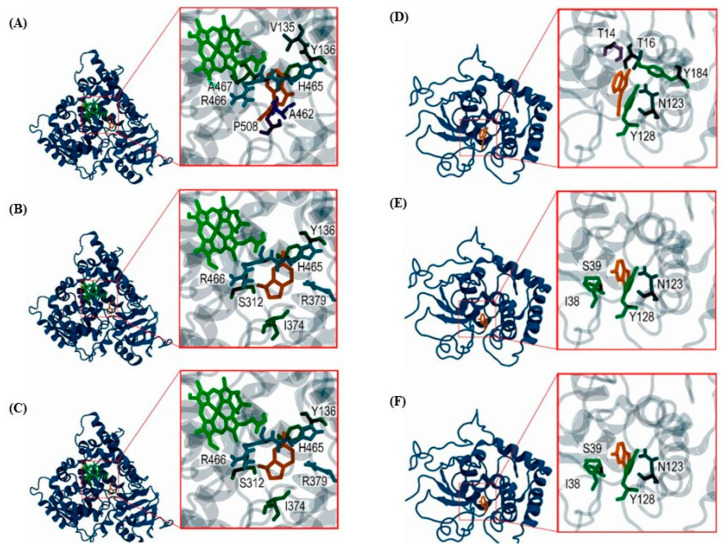
Three-dimensional representations of enzyme–ligand interactions for both CYP51 and GST enzymes. (**A**) CYP51_3-Indoleacetonitrile, (**B**) CYP51_Indole-3-Carboxaldehyde, and (**C**) CYP51_6-Methyl-1H-Indole. In these, the CYP51 enzyme is shown in blue, ligands in orange, and Heme B as a cofactor in green. (**D**) GST_3-Indoleacetonitrile, (**E**) GST_Indole-3-Carboxaldehyde, and (**F**) GST_6-Methyl-1H-Indole, where GST is visualized in blue and ligands in orange. The models were visualized using VMD software (version 1.9.3).

Analyzing the interacting residues for both CYP51 and GST enzymes with various ligands, including substrates, inhibitors, and GHPs, highlights key residues integral to enzyme functionality and ligand binding. In CYP51, the residues Y136, S312, and P508 are frequently involved in interactions across all ligand categories, suggesting their critical role in substrate recognition, binding, and inhibitor interaction. Additionally, residues Y122, P460, and A462 are also identified in CYP51 as contributing to the motifs and putative substrate recognition sites, as described in previous studies [43,44]. Similarly, in GST, the residues I38 and N123 are repeatedly involved in ligand interactions, indicating their importance in stabilizing enzyme–ligand complexes. Figure 3 illustrates the three-dimensional interactions between CYP51 and GST enzymes with indole GHPs, revealing the conformational details of the binding sites and the interaction with the previous residues. 

### 2.4. In Vitro Antifungal Activity of GHPs Against Botrytis cinerea: Mycelial Growth and Conidial Germination Inhibition

The in vitro antifungal activity of GHPs was investigated against *B. cinerea*. This study focused on evaluating the inhibitory effects of GHPs on two key developmental stages of the fungus: mycelial growth and conidial germination. 

**Figure 4:**
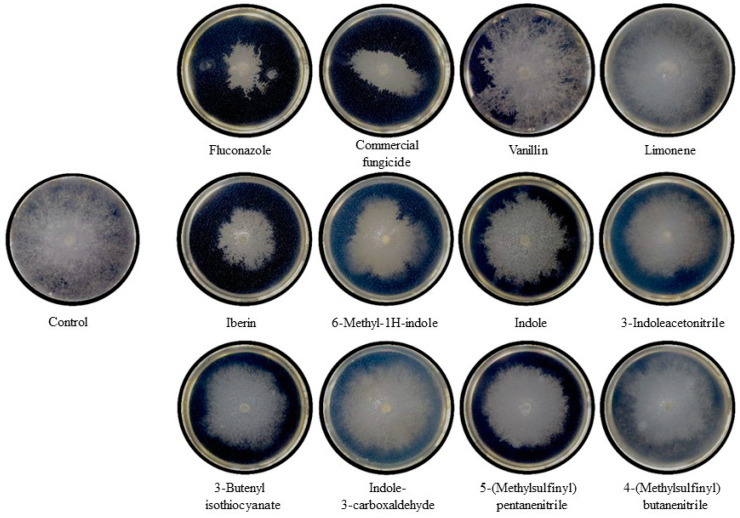
Comparative Analysis of Mycelial Growth Inhibition at 72 Hours. *B. cinerea* (strain CCCT 21.01) was centrally inoculated on PDA plates, with inhibitors or GHPs placed at the periphery. Zones of mycelial growth inhibition, shown as clear halos, were measured against a control with no inhibitors applied.

Figure 4 depicts a comparative study of mycelial growth inhibition induced by standard fungicides and GHPs against the fungal strain Botrytis cinerea. The images represent Petri dishes observed 72 h after the central inoculation of the pathogen onto potato dextrose agar (PDA) medium, with the application of standard fungicides or GHPs at the periphery of the plates. From left to right, the first row illustrates the effects of Fluconazole, a commercial fungicide (ANASAC^®^), Vanillin, and Limonene, while the second and the third rows show the inhibitory patterns of GHPs (Iberin, 6-Methyl-1H-indole, Indole, 3-Indoleacetonitrile, 3-Butenyl isothiocyanate, Indole-3-carboxaldehyde, 5-(Methylsulfinyl) pentanenitrile, and 4-(Methylsulfinyl) butanenitrile). Each compound’s efficacy is visually evident through the variation in mycelial coverage, with clear zones indicating strong inhibition. The control plate (top left) displays full mycelial coverage, providing a baseline for comparison.

Notably, Fluconazole, a widely recognized antifungal agent, and commercial fungicide shows significant mycelial suppression, serving as a comparative standard for the efficacy of GHPs. Natural compounds, such as Vanillin and Limonene, demonstrated modest inhibitory halos against mycelial expansion. In contrast, the GHPs uniformly revealed mycelial growth inhibition compared to the control. Specifically, the GHP Iberin led to the most pronounced inhibition, while Indole-3-carboxaldehyde displayed the least inhibitory effect. 

**Figure 5:**
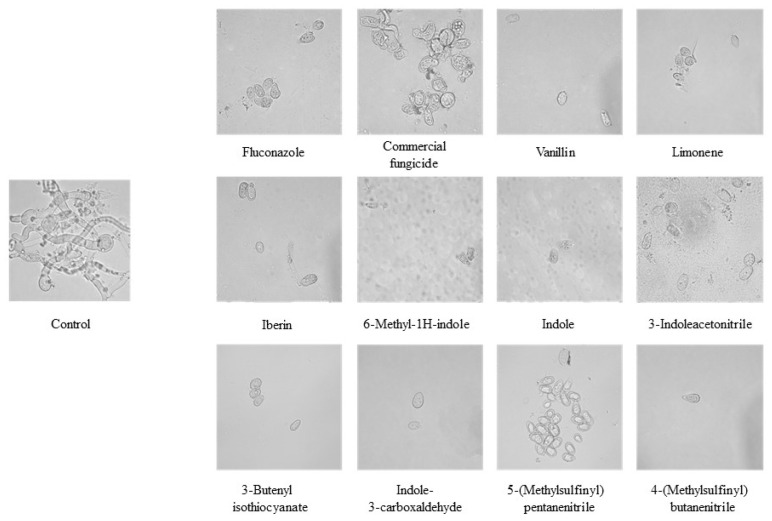
Optical microscopy view of conidial germination on PDA after 72 h of incubation at a magnification of 100×. The image shows the inhibitory growth percentage of different inhibitors or GHPs on conidial development.

Finally, investigating the antifungal potential of GHPs, the conidial germination assay was performed under controlled conditions. Figure 5 shows optical microscopy analysis of conidial germination at 72 h post-inoculation, magnified 100 times (100×) to capture the details of fungal propagation. The treatments, including standard fungicides and GHPs, were studied to evaluate their inhibitory effects on the germination process.

Optical microscopy analysis reveals that fluconazole, vanillin, and limonene inhibit conidial germination, whereas commercial fungicide allows hyphae formation and spore germination. In contrast, the eight tested GHPs suppress germination without hyphae formation, and some even induce cellular disruption, as evidenced in 6-methyl-1h-indole, Indole, and 3-Indoleacetonitrile. Notably, 5-(Methylsulfinyl) pentanenitrile, despite not permitting conidial germination, maintains conidial integrity, indicating a potent germination inhibition without causing degradation.

Additionally, spectrophotometric analysis was performed by measuring the optical density (OD) at 600 nm to estimate the germination and biomass growth of *B. cinerea* spores in liquid culture. Corroboration of the optical microscopy analyses, fluconazole, indole, 3-butenyl isothiocyanate, and 4-(methylsulfinyl) butanenitrile demonstrated an initial increase in absorbance for up to 12 h, after which the levels remained constant and ultimately lower than the control at 72 h. Moreover, decreases in absorbance over time were observed with commercial fungicides, vanillin, limonene, 5-(methylsulfinyl) pentanenitrile, iberin, and 6-methyl-1H-indole. These observations suggest cytological alterations, such as cytoplasmic granulation, retraction, and mycelial contraction, leading to the absorbance decrease [45].

## 3. Discussion

The identification of eight distinct GHPs in different tissues of broccoli via GC-MS analysis expands our understanding of the complex biochemical profiles of cruciferous vegetables and their potential roles in plant defense mechanisms. The characterization of these compounds into aliphatic and indolic groups highlights their varied chemical structures and potential roles in plant physiology and defense mechanisms. Sulforaphane nitrile was notably identified as the predominant GHP in the inflorescences and stems, suggesting its major role in the plant’s defense strategy against pathogens, as supported by its significant representation in peak area percentage. This aligns with previous research indicating glucoraphanin as the most abundant glucosinolate in broccoli, further emphasizing the defensive importance of its hydrolysis products [23,31].

It is notable that no strictly aromatic GHPs were found in the analysis, underscoring the distinct composition of glucosinolate breakdown products in broccoli [46,47]. These findings highlight the distinct biochemical properties, the diverse biological activities, and potential applications of these GHPs in agricultural applications [48,49]. Their use as natural biocontrol agents offers the potential for eco-friendly and economical crop protection strategies. These eight GHPs could play a crucial role in controlling fungal infections, particularly against the phytopathogenic Botrytis cinerea [4].

The strategic selection of 14-alpha-demethylase (CYP51, EC 1.14.14.154) and glutathione-S-transferase (GST, EC 2.5.1.18) in our docking studies highlights their pivotal roles in the detoxification processes of *B. cinerea*. CYP51 is involved in the biosynthesis of ergosterol, a vital component of fungal cell membranes. Inhibiting this enzyme disrupts cell membrane integrity, leading to fungal cell death [1]. Similarly, GST is integral to the detoxification of reactive oxygen species and xenobiotics through conjugation with glutathione [2]. Targeting these enzymes with glucosinolate hydrolysis products (GHPs) leverages their detoxifying functions to develop effective antifungal strategies. Our molecular-docking studies revealed significant interactions between GHPs and these enzymes, with indolic GHPs showing substantial stability, particularly involving residues Y136 in CYP51 and N123 in GST. This suggests their potential as competitive inhibitors, aligning with observed antifungal activity against *B. cinerea* [3,4].

The successful modeling of CYP51 and GST enzymes using the I-TASSER approach was significant, especially given the lower sequence identities with known structures. This suggests that the modeling approach employed by I-TASSER was primarily ab initio threading rather than homology-based modeling, a technique typically reserved for instances where sequence identities fall below the 70% threshold [50]. The QMEANDisCo Global score of 0.77 ± 0.05 for CYP51, along with the ERRAT Overall Quality Factor of 94.2797, indicates a high-quality model. Despite these positive evaluations, PROCHECK analysis revealed six errors. Therefore, to further improve the model, molecular-dynamics (MD) simulations were performed to relax the bonds and refine the structure. Similarly, the GST model, with a QMEANDisCo Global score of 0.66 ± 0.05 and ERRAT Overall Quality Factor of 82.9268, demonstrated moderate quality. Given the seven errors and two warnings identified by PROCHECK, MD simulations were conducted to enhance the model by relaxing the bonds and refining the structure.

The stability and variability in the RMSD values observed during the molecular-dynamics simulations highlight the structural integrity and dynamic nature of these enzyme models. The determination of productive phases is crucial for understanding the structural stability and dynamic behavior of enzymes. Our 200 ns MD simulations demonstrated convergence in the range of 0.3 to 0.6 nm (3–6 Å), which is within the reported range for complex biological macromolecules, such as enzymes with cofactors. For CYP51, the relative stability in RMSD suggests a robust structure, which aligns well with previously reported structures, suggesting a similarity between the simulated and original structures [51,52]. In contrast, the fluctuating RMSD of the GST model during the initial simulation phases suggests a flexible enzyme structure, which might affect its interaction with various substrates and inhibitors, highlighting its potential vulnerability or adaptability in response to environmental stresses [53]. For instance, microsecond simulations of CYP2D6 variants did not achieve significant RMSD stability, indicating that even extended simulation times do not always result in complete stability [34]. This lack of stability can be attributed to the structural flexibility and dynamic nature of its active site. GST enzymes are known to interact with multiple substrates and undergo significant conformational changes, leading to RMSD fluctuations [54,55]. These findings are consistent with reports on the behavior of apo and holo enzyme complexes in MD simulations, where the presence or absence of substrates or cofactors significantly impacts enzyme stability [56,57]. Our findings suggest that the productive phases for CYP51 and GST are within the expected range for complex enzymatic structures. The deposition of these models into the PMDB and their subsequent use in molecular-docking studies aim to advance our understanding of how these enzymes interact with glucosinolate hydrolysis products.

The successful application of I-TASSER to model these structures and the subsequent validation through molecular dynamics simulations provide a promising methodological framework for further research. Furthermore, the docking studies reveal the intricate interactions between GHPs and fungal enzymes, with indolic GHPs showing substantial stability in their interactions. The analysis of interacting residues, especially the recurring involvement of Y136 in CYP51 and N123 in GST, with GHPs, underscore their potential as targets for enzyme modulation [42]. This observation is particularly significant as it suggests that indolic GHPs could serve as competitive inhibitors, a hypothesis that aligns with their observed antifungal activity against *B. cinerea*. 

In this study, the antifungal activity of GHPs against *B. cinerea* was demonstrated through both in silico and in vitro experiments. Molecular-docking simulations revealed significant interactions between GHPs and the detoxifying enzymes, suggesting their potential as competitive inhibitors. The in vitro assays further confirmed the antifungal efficacy of these compounds, indicating their promise as eco-friendly crop protection agents.

Future research should focus on elucidating the detailed mechanisms of action of GHPs and assessing their efficacy and safety in field conditions. 

## 4. Materials and Methods

### 4.1. Plant Materials

Broccoli inflorescences, leaves, and stems were obtained from a local market and immediately transported to the laboratory. Upon arrival, the samples were treated with liquid nitrogen to freeze them. Subsequently, the samples were stored at −80 °C until further analysis. 

### 4.2. Preparation, Extraction and Analysis of Glucosinolate Hydrolysis Products (GHPs)

One gram of plant material (leaf, stem, or inflorescence) previously pulverized with liquid nitrogen was weighed and placed in a 100 mL glass flask. Then, 50 mL of 100 mM sodium phosphate buffer (pH 7), supplemented with 0.01 mM iron (II) sulfate (FeSO_4_), was added. The solution was incubated in a thermostatically controlled bath at 25 °C for 1 h to facilitate the hydrolysis of glucosinolates [58,59]. After the incubation period, 50 mL of dichloromethane was added to the sample, and vigorous agitation was maintained for 30 min to facilitate the extraction of GHPs from the plant tissue. The sample was then subjected to phase separation using a separatory funnel, where the organic phase, rich in GHPs, was collected and filtered through vacuum filtration to remove plant debris. Subsequently, the extract was concentrated to complete dryness using rotary evaporation, and then resuspended in 1 mL of HPLC-grade dichloromethane and filtered using a 0.22 μm PVDF syringe filter to remove any particulate matter or impurities [60].

Finally, GHPs were analyzed using Gas Chromatography-Mass Spectrometry/Mass Spectrometry (GC-MS/MS) on a Perkin Elmer Clarus 680 system, coupled with a mass spectrometry detector Clarus SQ 8T (PerkinElmer, Waltham, MA, USA). The analysis was performed using an Agilent HP-5MS column (30 m × 0.25 mm × 0.25 µm) with specific analytical conditions (Agilent Technologies, Santa Clara, CA, USA). The temperature program for the analysis was based on the method described in the literature [23,61]. Initially, the column temperature was set at 35 °C for 3 min, followed by an increase to 280 °C at a rate of 12 °C per minute, then further increased to 310 °C at a rate of 30 °C per minute and held for 3 min. The injector and detector temperatures were set at 200 °C and 300 °C, respectively. Helium was used as the carrier gas at a flow rate of 25 mL per minute. The GHPs were identified by analyzing their mass spectra and retention times, using the NIST database for compound identification (Figure S2).

### 4.3. In Silico Analysis of GHP Interactions with B. cinerea Enzymes

To investigate the molecular mechanism underlying the antifungal activity of GHPs against *B. cinerea*, molecular interaction simulations were performed using AutoDock Vina. These simulations specifically focused on the interactions between the GHPs and two detoxifying enzymes of *B. cinerea*, namely eburicol 14-alpha-demethylase (CYP51 (EC 1.14.14.154)) and glutathione-S-transferase (GST (EC 2.5.1.18)). 

#### 4.3.1. Protein Structure Retrieval and Dynamic Modeling of CYP51 and GST

The amino acid sequences of the enzymes were obtained from the UniProtKB database (https://www.uniprot.org/, last accessed 25 March 2023) [62], with CYP51 identified under UniProt Code: Q9P428 and GST under UniProt Code: Q9HF89. Five three-dimensional models of the enzymes of interest were obtained using the I-TASSER server (https://zhanggroup.org/I-TASSER/, last accessed 8 June 2023) [50]. The models with the highest C-score and TM-score values, indicating high prediction quality, were selected, and their topology was described. Various server tools to validate the 3D models of CYP51 and GST were used, including QMEAN [63], SAVESv6.0 (Errat, Verify3D, Procheck) [64,65,66,67], ProSA [68], and PDBsum [69]. Due to technical issues, ANOLEA and MolProbity were not utilized. The Clustal Omega tool [70] (https://www.ebi.ac.uk/Tools/msa/clustalo/, last accessed 8 June 2023) was utilized to perform a multiple sequence alignment of the enzymes with the templates used by I-TASSER to define the modeling method. GROMACS 2020.3 software [71] (University of Groningen, Groningen, The Netherlands) was employed to evaluate the stability and dynamic properties of the selected three-dimensional models through 200 ns molecular dynamics simulations. The root mean square deviation (RMSD) values were analyzed to identify energetically stable conformations. The time frame displaying the most stable RMSD values was selected to obtain the refined models along with their topology.

#### 4.3.2. Molecular Docking of GHPs with *B. cinerea* CYP51 and GST

Substrates and inhibitors of the target enzymes, CYP51 and GST, were searched in the BRENDA servers (https://www.brendaenzymes.org/, last accessed 8 April 2023) [72]. To determine the binding site of each enzyme, glutathione and obtusifoliol were employed as reference ligands for GST and CYP51, respectively. For inhibitory interaction, diniconazole, limonene, triadimefon, vanillin, carbendazim, tebuconazole, and fluconazole, were simulated [40,41,42]. The hydrolysis products of glucosinolates (GHPs) considered for the simulations were as follows: 3-indolacetonitrile; 4-(methylsulfinyl) butanenitrile; 6-methyl-1H-indole; iberin; indole; indole-3-carboxaldehyde; 3-butenyl isothiocyanate; and 5-(methylsulfinyl) pentanenitrile. Molecular docking was performed using the AutoDock Vina 1.2.2 software via ADT [73] (National Institutes of Health, Bethesda, MD, USA), utilizing the refined three-dimensional models and ligand structures obtained from the PubChem database (https://pubchem.ncbi.nlm.nih.gov/, last accessed 8 April 2023) [74]. The specific grid parameters used were as follows: for CYP51, a grid size of 126 × 126 × 126 with center coordinates (83.191; 81.255; 85.359), and for GST, a grid size of 58 × 88 × 64 with center coordinates (50.191; 64.255; 66.359), both with a spacing of 0.36 Å. The exhaustiveness parameter was set to 8 to ensure thorough sampling of the binding modes. The affinity energies for the ligand–enzyme interactions were calculated based on the lowest energy conformations [75]. three-dimensional structures were visualized using PyMOL (version 2.3.0), developed by Schrödinger, LLC, (New York, NY, USA) [76].

### 4.4. In Vitro Analysis of GHPs against B. cinerea

#### 4.4.1. Preparation of GHPs and Cultivation of *Botrytis cinerea*

Eight synthesized pure GHPs, previously identified in leaf, stem, or inflorescence, were prepared at a 50 mM concentration using organic solvents as per manufacturer guidelines (Molport^®^, Vilnius, Lithuania, 2020). Additionally, to serve as positive controls in this study, fluconazole, limonene, and a commercially available fungicide, Fungicida Hongos Anasac (Anasac, Santiago, Chile, 2018) were also prepared at the same concentration. 

The CCCT 21.01 strain of *Botrytis cinerea*, obtained from the Colección Chilena de Cultivos Tipo (Universidad de la Frontera, Temuco, Chile, 2020), was used in this study. A sterile 0.5 cm mycelial disc was aseptically transferred onto Petri dishes containing Potato Dextrose Agar (PDA) medium, composed of 2.4% potato dextrose and 2.0% agar. The inoculated plates were sealed and placed in a controlled environment at 25 °C for 4 days to facilitate proper growth. Following incubation, the plates were refrigerated, serving as the working stock for subsequent assays.

#### 4.4.2. Evaluation of the Antifungal Activity of GHPs

The effects of the GHPs on *B. cinerea* were assessed using mycelial growth inhibition and inhibition of conidial germination assays. Inhibition of *B. cinerea* fungal mycelial growth was evaluated using Petri dishes containing Potato Dextrose Agar (PDA) medium. A 0.5 cm mycelial disc of *B. cinerea* was inoculated at the center of each plate, while 5 µL of the experimental solution being tested was added to the right and left ends of the plate. The plates were then incubated at 25 °C. Photographic documentation was conducted every 24 h until the mycelium on the control plate occupied the entire surface [77,78,79,80]. The images were processed using Adobe Photoshop 2021 and analyzed using the Image J Fiji software (version 1.53c, https://imagej.net/Fiji, National Institutes of Health, Bethesda, MD, USA). The mycelial area was measured in pixels and then converted to cm^2^. Inhibition of conidial germination of *B. cinerea* was assessed using multi-well cell culture plates with PD medium (2.4% potato dextrose). To initiate the experiments, *B. cinerea* spores were collected and quantified using a Neubauer chamber, adjusting the concentration to 2x10^5^ (spores/mL). A total of 150 µL of spore suspension was mixed with 50 µL of the experimental solution being tested and inoculated onto the plates. As controls, plates containing 150 µL of spores and 50 µL of medium were incubated, while plates with 150 µL of medium and 50 µL of the experimental solution were used as blanks. The plates were incubated at 20 °C for 72 h, and the absorbance at 490 nm was measured every 12 h. Changes in optical density were used to determine the growth of *B. cinerea* [45,78,80]. 

## 5. Conclusions

This study has revealed the antifungal properties of glucosinolate hydrolysis products (GHPs) derived from broccoli by-products, demonstrating a spectrum of antifungal effectiveness that underscores their potential as biocontrol agents. The efficacy of these GHPs, particularly indolic compounds, in inhibiting both mycelial growth and conidial germination of *Botrytis cinerea* positions them as competitive inhibitors of the fungus’s detoxifying enzymes, offering a sustainable and eco-friendly alternative to conventional fungicides through the utilization of agricultural residues. Further research is imperative to elucidate the mechanisms of action of GHPs and to assess their practical applications in controlling fungal pathogens. Such studies are crucial for advancing our understanding and development of novel biofungicides, thereby contributing to the establishment of more environmentally sustainable and effective strategies for plant disease management.

## Figures and Tables

**Table 1:** Identification of glucosinolates hydrolysis products (GHP) detected in broccoli inflorescences, leaves, and stems by GC-MS with the corresponding retention times (t_R_) and peak areas (% of total).

Peak	GHP Compound Name	Molecular Formula	Molecular Weight (g/mol)	t_R_. (min)	Peak Area (% of Total)		
					Inflorescences	Leaves	Steams
1	1-butene, 4-isothiocyanato-	C_5_H_7_NS	113	9.29	4.03	1.19	4.92
2	Indole	C_8_H_7_N	117	13.22	9.23	8.37	5.5
3	Iberin Nitrile	C_5_H_9_NOS	131	13.98	4.39	n.d. ^1^	3.56
4	5-methylindole	C_9_H_9_N	131	14.01	4.29	5.42	4.88
5	Sulforaphane nitrile	C_6_H_11_NOS	145	15.04	33.8	9.14	46.88
6	Iberin	C_5_H_9_NOS_2_	163	15.74	7.08	54.39	14.85
7	1H-Indole-3-acetonitrile	C_10_H_8_N_2_	156	16.96	22.96	17.04	12.85
8	1H-Indole-3-carboxaldehyde	C_9_H_7_NO	145	17.02	14.22	4.45	6.56

^1^ n.d.: none detected.

**Table 2:** Affinity energy values (kcal/mol) determined from the Autodock Vina program, for substrates, inhibitors, and eight glucosinolate hydrolysis products (GHPs) interacting with Botrytis cinerea CYP51 and GST enzymes.

Ligand Type	Ligand Name	Affinity Energy (kcal/mol)		Interacting Residues	
		CYP51	GST	CYP51	GST
Substrates	Glutathione	-	−6.7		N16; K19; R56; I57; F73; S75; G120; Q121
	Obtusifoliol	−3.5	-	E119; E120; D137; P139	
Inhibitors	Carbendazim	−6.7	−6.6	Y136; H311; S312; S313; C467; P508	N16; K19; I57; Q119; G120; N123; W178; I181
	Diniconazole	−4.6	−6.8	Y122; V135; Y136; A308; S312; I374; R379; F460; G461; A462; H465; R466; C467; P508	T12; N16; K19; I38; I57; G120; N123; W178
	Fluconazole	−8.3	−8.2	Y122; Y136; S312; I374; P459; F460; A462; H465; R466; C467; P508	N16; I38; R56; I57; G120; N123; P128
	Limonene	−6.1	−5.2	Y122; Y136; S312; R379; A462; H465; P508	I38; N123
	Tebuconazole	−7.0	−7.2	Y122; L125; Y136; F234; I374; S376; F460; G461; A462; R466; L507; P508	T14; K19; I38; R56; I57; P58; G120; N123; H124; W178
	Triadimefon	−6.4	−6.3	V135; Y136; A308; S312; I374; I377; F460; G461; A462; R466; C467; P508	T12; T14; I38; NT123; PT128
	Vanillin	−5.8	−5.3	Y122; S312; R379; A462; P508	T14; I38; Q121; Y128
GHPs	3-Indoleacetonitrile	−6.4	−6.4	V135; Y136; A462; H465; R466; C467; P508	T14; N16; N123; Y128; Y184
	4-(Methylsulfinyl) butane nitrile	−4.3	−4.2	S312	T12; I38; Y128
	6-Methyl-1H-indole	−5.5	−5.3	Y136; S312; I374; R379; HT65; R466	I38; S39; N123; Y128
	Iberin	−4.1	−3.8	Y122; F460	I57; NT1T23; HT124
	Indole	−5.7	−5.2	Y122; Y136; I374; S376; A462; L507; P508	K19; G120; N123; W178
	Indole-3-carboxaldehyde	−6.2	−5.7	Y136; S312; I374; F460; A462; P508	I38; N123
	3-Butenyl isothiocyanate	−4.0	−3.7	Y122; R379; L507	I38; G120; N123
	5-(Methylsulfinyl) pentanenitrile	−4.7	−4.2	Y122; S312; I374; F460	T12; N123; Y128

## Data Availability

The data presented in this study are available on request from the corresponding author.

## Supplementary Materials

The following supporting information can be downloaded at: https://www.mdpi.com/article/10.3390/ijms25147945/s1.

## References

[1] Jaffar N.S., Jawan R., Chong K.P. (2023). The potential of lactic acid bacteria in mediating the control of plant diseases and plant growth stimulation in crop production—A mini review. Front. Plant Sci..

[2] Xu X., Chen Y., Li B., Zhang Z., Qin G., Chen T., Tian S. (2022). Molecular mechanisms underlying multi-level defense responses of horticultural crops to fungal pathogens. Hortic. Res..

[3] Matrose N.A., Obikeze K., Belay Z.A., Caleb O.J. (2021). Plant extracts and other natural compounds as alternatives for post-harvest management of fruit fungal pathogens: A review. Food Biosci..

[4] Buxdorf K., Yaffe H., Barda O., Levy M. (2013). The Effects of Glucosinolates and Their Breakdown Products on Necrotrophic Fungi. PLoS ONE.

[5] Ribes S., Fuentes A., Talens P., Barat J.M. (2018). Prevention of fungal spoilage in food products using natural compounds: A review. Crit. Rev. Food Sci. Nutr..

[6] Ugolini L., Martini C., Lazzeri L., D’Avino L., Mari M. (2014). Control of postharvest grey mould (Botrytis cinerea Per.: Fr.) on strawberries by glucosinolate-derived allyl-isothiocyanate treatments. Postharvest Biol. Technol..

[7] Andriana Y., Fajriani N.A., Iwansyah A.C., Xuan T.D. (2023). Phytochemical Constituents of Indonesian Adlay (*Coix lacrima-jobi* L.) and Their Potential as Antioxidants and Crop Protection Agents. Agrochemicals.

[8] Sciubba F., Chronopoulou L., Pizzichini D., Lionetti V., Fontana C., Aromolo R., Socciarelli S., Gambelli L., Bartolacci B., Finotti E. (2020). Olive Mill Wastes: A Source of Bioactive Molecules for Plant Growth and Protection against Pathogens. Biology.

[9] Del Carmen Martínez-Ballesta M., Moreno D.A., Carvajal M. (2013). The Physiological Importance of Glucosinolates on Plant Response to Abiotic Stress in Brassica. Int. J. Mol. Sci..

[10] Thomas M., Badr A., Desjardins Y., Gosselin A., Angers P. (2018). Characterization of industrial broccoli discards (*Brassica oleracea* var. italica) for their glucosinolate, polyphenol and flavonoid contents using UPLC MS/MS and spectrophotometric methods. Food Chem..

[11] Lelario F., Bianco G., Bufo S.A., Cataldi T.R.I. (2012). Establishing the occurrence of major and minor glucosinolates in Brassicaceae by LC–ESI-hybrid linear ion-trap and Fourier-transform ion cyclotron resonance mass spectrometry. Phytochemistry.

[12] Sønderby I.E., Geu-Flores F., Halkier B.A. (2010). Biosynthesis of glucosinolates--gene discovery and beyond. Trends Plant Sci..

[13] Ladak Z., Khairy M., Armstrong E.A., Yager J.Y., Ghosh D. (2021). Chapter 11—Glucosinolates: Paradoxically beneficial in fighting both brain cell death and cancer. Nutraceuticals in Brain Health and Beyond.

[14] Bhat R., Kour J., Nayik G.A. (2022). Chapter 12—Glucosinolates. Nutraceuticals and Health Care.

[15] Ahuja I., de Vos R.C.H., Bones A.M., Hall R.D. (2010). Plant molecular stress responses face climate change. Trends Plant Sci..

[16] Zhu B., Liang Z.L., Zang Y.X., Zhu Z.J., Yang J. (2023). Diversity of glucosinolates among common Brassicaceae vegetables in China. Hortic. Plant J..

[17] Latté K.P., Appel K.E., Lampen A. (2011). Health benefits and possible risks of broccoli—An overview. Food Chem. Toxicol..

[18] Piasecka A., Jedrzejczak-Rey N., Bednarek P. (2015). Secondary metabolites in plant innate immunity: Conserved function of divergent chemicals. New Phytol..

[19] Aghajanzadeh T.A., Watanabe M., Tohge T., Hawkesford M.J., Fernie A.R., Hoefgen R., Elzenga J.T.M., De Kok L.J. (2023). Necrotrophic fungal infection affects indolic glucosinolate metabolism in *Brassica rapa*. Acta Physiol. Plant..

[20] Madloo P., Lema M., Cartea M.E., Soengas P. (2021). *Sclerotinia sclerotiorum* Response to Long Exposure to Glucosinolate Hydrolysis Products by Transcriptomic Approach. Microbiol. Spectr..

[21] Ayaz M., Ullah F., Sadiq A., Ullah F., Ovais M., Ahmed J., Devkota H.P. (2019). Synergistic interactions of phytochemicals with antimicrobial agents: Potential strategy to counteract drug resistance. Chem. Biol. Interact..

[22] Angelino D., Dosz E.B., Sun J., Hoeflinger J.L., Van Tassell M.L., Chen P., Harnly J.M., Miller M.J., Jeffery E.H. (2015). Myrosinase-dependent and –independent formation and control of isothiocyanate products of glucosinolate hydrolysis. Front. Plant Sci..

[23] Román J., González D., Inostroza-Ponta M., Mahn A. (2020). Molecular Modeling of Epithiospecifier and Nitrile-Specifier Proteins of Broccoli and Their Interaction with Aglycones. Molecules.

[24] Albertini C., Thebaud G., Fournier E., Leroux P. (2002). Eburicol 14α-demethylase gene (CYP51) polymorphism and speciation in Botrytis cinerea. Mycol. Res..

[25] Zhang C., Imran M., Liu M., Li Z., Gao H., Duan H., Zhou S., Liu X. (2020). Two Point Mutations on CYP51 Combined With Induced Expression of the Target Gene Appeared to Mediate Pyrisoxazole Resistance in Botrytis cinerea. Front. Microbiol..

[26] Zhang C., Li T., Xiao L., Zhou S., Liu X. (2020). Characterization of tebuconazole resistance in Botrytis cinerea from tomato plants in China. Phytopathol. Res..

[27] Vanduchova A., Anzenbacher P., Anzenbacherova E. (2019). Isothiocyanate from Broccoli, Sulforaphane, and Its Properties. J. Med. Food.

[28] Li H., Xia Y., Liu H.Y., Guo H., He X.Q., Liu Y., Wu D.T., Mai Y.H., Li H.B., Zou L. (2022). Nutritional values, beneficial effects, and food applications of broccoli (*Brassica oleracea* var. italica Plenck). Trends Food Sci. Technol..

[29] Li Z.S., Zheng S.N., Liu Y.M., Fang Z.Y., Yang L.M., Zhuang M., Zhang Y.Y., Lv H.H., Wang Y., Xu D.H. (2021). Characterization of glucosinolates in 80 broccoli genotypes and different organs using UHPLC-Triple-TOF-MS method. Food Chem..

[30] Villano D., Fernandez-Pan I., Arozarena I., Ibanez F.C., Virseda P., Beriain M.J. (2023). Revalorisation of broccoli crop surpluses and field residues: Novel ingredients for food industry uses. Eur. Food Res. Technol..

[31] Liu M., Zhang L., Ser S.L., Cumming J.R., Ku K.M. (2018). Comparative Phytonutrient Analysis of Broccoli By-Products: The Potentials for Broccoli By-Product Utilization. Molecules.

[32] Gudino I., Martin A., Casquete R., Prieto M.H., Ayuso M.C., Cordoba M.G. (2022). Evaluation of broccoli (*Brassica oleracea* var. italica) crop by-products as sources of bioactive compounds. Sci. Hortic..

[33] Silva R.C., Freitas H.F., Campos J.M., Kimani N.M., Silva C., Borges R.S., Pita S.S.R., Santos C.B.R. (2021). Natural Products-Based Drug Design against SARS-CoV-2 Mpro 3CLpro. Int. J. Mol. Sci..

[34] Don C.G., Smieško M. (2018). Microsecond MD simulations of human CYP2D6 wild-type and five allelic variants reveal mechanistic insights on the function. PLoS ONE.

[35] Moriwaki Y., Terada T., Tsumoto K., Shimizu K. (2015). Rapid Heme Transfer Reactions between NEAr Transporter Domains of Staphylococcus aureus: A Theoretical Study Using QM/MM and MD Simulations. PLoS ONE.

[36] Kandeel M., Kitade Y., Al-Taher A., Al-Nazawi M. (2019). The structural basis of unique substrate recognition by Plasmodium thymidylate kinase: Molecular dynamics simulation and inhibitory studies. PLoS ONE.

[37] Basnet S., Marahatha R., Shrestha A., Bhattarai S., Katuwal S., Sharma K.R., Marasini B.P., Dahal S.R., Basnyat R.C., Patching S.G. (2022). In Vitro and In Silico Studies for the Identification of Potent Metabolites of Some High-Altitude Medicinal Plants from Nepal Inhibiting SARS-CoV-2 Spike Protein. Molecules.

[38] Dixon D.P., Lapthorn A., Edwards R. (2002). Plant glutathione transferases. Genome Biol..

[39] Hargrove T.Y., Kim K., de Nazaré Correia Soeiro M., da Silva C.F., Batista D.D., Batista M.M., Yazlovitskaya E.M., Waterman M.R., Sulikowski G.A., Lepesheva G.I. (2012). CYP51 structures and structure-based development of novel, pathogen-specific inhibitory scaffolds. Int. J. Parasitol. Drugs Drug Resist..

[40] Mathieu Y., Prosper P., Buée M., Dumarçay S., Favier F., Gelhaye E., Gérardin P., Harvengt L., Jacquot J.P., Lamant T. (2012). Characterization of a Phanerochaete chrysosporium glutathione transferase reveals a novel structural and functional class with ligandin properties. J. Biol. Chem..

[41] Ming Z.-H., Xu S.-Z., Zhou L., Ding M.-W., Yang J.-Y., Yang S., Xiao W.-J. (2009). Organocatalytic synthesis and sterol 14α-demethylase binding interactions of enantioriched 3-(1H-1,2,4-triazol-1-yl)butyl benzoates. Bioorganic Med. Chem. Lett..

[42] Meux E., Morel M., Lamant T., Gérardin P., Jacquot J.-P., Dumarçay S., Gelhaye E. (2013). New substrates and activity of Phanerochaete chrysosporium Omega glutathione transferases. Biochimie.

[43] Lepesheva G.I., Waterman M.R. (2007). Sterol 14alpha-demethylase cytochrome P450 (CYP51), a P450 in all biological kingdoms. Biochim. Biophys. Acta.

[44] Zhang J., Li L., Lv Q., Yan L., Wang Y., Jiang Y. (2019). The Fungal CYP51s: Their Functions, Structures, Related Drug Resistance, and Inhibitors. Front. Microbiol..

[45] Reglinski T., Elmer P., Taylor J., Wood P., Hoyte S. (2010). Inhibition of Botrytis cinerea growth and suppression of botrytis bunch rot in grapes using chitosan. Plant Pathol..

[46] Guo Y., Wang L., Chen Y., Yun L., Liu S., Li Y. (2018). Stalk length affects the mineral distribution and floret quality of broccoli (*Brassica oleracea* L. var. italica) heads during storage. Postharvest Biol. Technol..

[47] Salas-Millán J.-Á., Aznar A., Conesa E., Conesa-Bueno A., Aguayo E. (2022). Functional food obtained from fermentation of broccoli by-products (stalk): Metagenomics profile and glucosinolate and phenolic compounds characterization by LC-ESI-QqQ-MS/MS. LWT.

[48] Jeschke V., Gershenzon J., Vassao D.G., Kopriva S. (2016). Insect Detoxification of Glucosinolates and Their Hydrolysis Products. Glucosinolates.

[49] Hopkins R.J., van Dam N.M., van Loon J.J. (2009). Role of glucosinolates in insect-plant relationships and multitrophic interactions. Annu. Rev. Entomol..

[50] Yang J., Yan R., Roy A., Xu D., Poisson J., Zhang Y. (2015). The I-TASSER Suite: Protein structure and function prediction. Nat. Methods.

[51] Çevik U.A., Celik I., Işık A., Pillai R.R., Tallei T.E., Yadav R., Özkay Y., Kaplancıklı Z.A. (2022). Synthesis, molecular modeling, quantum mechanical calculations and ADME estimation studies of benzimidazole-oxadiazole derivatives as potent antifungal agents. J. Mol. Struct..

[52] Shi N., Zheng Q., Zhang H. (2020). Molecular Dynamics Investigations of Binding Mechanism for Triazoles Inhibitors to CYP51. Front. Mol. Biosci..

[53] Gao Q., Lu C., Wang X.W., Zhang J.W., Song Y., Xue Y.L. (2018). Molecular dynamics simulation and steered molecular dynamics simulation on irisin dimers. J. Mol. Model..

[54] Omae Y., Saito H., Takagi H., Nishimura M., Iwayama M., Kawaguchi K., Nagao H. (2012). Molecular Dynamics Study of Glutathione. Quantum Systems in Chemistry and Physics.

[55] Stella L., Di Iorio E.E., Nicotra M., Ricci G. (1999). Molecular dynamics simulations of human glutathione transferase P1-1: Conformational fluctuations of the apo-structure. Proteins.

[56] de Souza T.L.F., Sanches D., Gonçalves R.B., da RochaPita S.S., Pascutti P.G., Bianconi M.L., de Almeida F.C.L., Silva J.L., de Oliveira A.C. (2010). Conformational selection, dynamic restriction and the hydrophobic effect coupled to stabilization of the BIR3 domain of the human X-linked inhibitor of apoptosis protein by the tetrapeptide AVPI. Biophys. Chem..

[57] Da Rocha Pita S.S., Batista P.R., Albuquerque M.G., Pascutti P.G. (2012). Molecular Dynamics Simulations of Peptide Inhibitors Complexed With Trypanosoma cruzi Trypanothione Reductase. Chem. Biol. Drug Des..

[58] Román J., Castillo A., Cottet L., Mahn A. (2018). Kinetic and structural study of broccoli myrosinase and its interaction with different glucosinolates. Food Chem..

[59] Liang H., Yuan Q.P., Dong H.R., Liu Y.M. (2006). Determination of sulforaphane in broccoli and cabbage by high-performance liquid chromatography. J. Food Compos. Anal..

[60] Kissen R., Bones A.M. (2009). Nitrile-specifier proteins involved in glucosinolate hydrolysis in Arabidopsis thaliana. J. Biol. Chem..

[61] Lee Y.S., Ku K.M., Becker T.M., Juvik J.A. (2017). Chemopreventive glucosinolate accumulation in various broccoli and collard tissues: Microfluidic-based targeted transcriptomics for by-product valorization. PLoS ONE.

[62] UniProt-Consortium (2021). UniProt: The universal protein knowledgebase in 2021. Nucleic Acids Res.

[63] Benkert P., Biasini M., Schwede T. (2011). Toward the estimation of the absolute quality of individual protein structure models. Bioinformatics.

[64] Colovos C., Yeates T.O. (1993). Verification of protein structures: Patterns of nonbonded atomic interactions. Protein Sci..

[65] Lüthy R., Bowie J.U., Eisenberg D. (1992). Assessment of protein models with three-dimensional profiles. Nature.

[66] Pontius J., Richelle J., Wodak S.J. (1996). Deviations from standard atomic volumes as a quality measure for protein crystal structures. J. Mol. Biol..

[67] Laskowski R.A., MacArthur M.W., Moss D.S., Thornton J.M. (1993). PROCHECK: A program to check the stereochemical quality of protein structures. J. Appl. Crystallogr..

[68] Wiederstein M., Sippl M.J. (2007). ProSA-web: Interactive web service for the recognition of errors in three-dimensional structures of proteins. Nucleic Acids Res..

[69] Laskowski R.A. (2009). PDBsum new things. Nucleic Acids Res..

[70] Sievers F., Wilm A., Dineen D., Gibson T.J., Karplus K., Li W., Lopez R., McWilliam H., Remmert M., Söding J. (2011). Fast, scalable generation of high-quality protein multiple sequence alignments using Clustal Omega. Mol. Syst. Biol..

[71] GROMACS d.t. (2020). GROMACS Documentation. Release 2020.3.

[72] Chang A., Jeske L., Ulbrich S., Hofmann J., Koblitz J., Schomburg I., Neumann-Schaal M., Jahn D., Schomburg D. (2021). BRENDA, the ELIXIR core data resource in 2021: New developments and updates. Nucleic Acids Res..

[73] Eberhardt J., Santos-Martins D., Tillack A.F., Forli S. (2021). AutoDock Vina 1.2.0: New Docking Methods, Expanded Force Field, and Python Bindings. J. Chem. Inf. Model..

[74] Kim S., Chen J., Cheng T., Gindulyte A., He J., He S., Li Q., Shoemaker B.A., Thiessen P.A., Yu B. (2023). PubChem 2023 update. Nucleic Acids Res.

[75] Trott O., Olson A.J. (2010). AutoDock Vina: Improving the speed and accuracy of docking with a new scoring function, efficient optimization, and multithreading. J. Comput. Chem..

[76] Schrödinger L., DeLano W. (2020). PyMOL. http://www.pymol.org/pymol.

[77] Ahn S.Y., Kim S.A., Yun H.K. (2015). Inhibition of Botrytis cinerea and accumulation of stilbene compounds by light-emitting diodes of grapevine leaves and differential expression of defense-related genes. Eur. J. Plant Pathol..

[78] Jeong M.-J., Bae D., Bae H., Lee S.I., Kim J.A., Shin S.C., Park S.H., Park S.-C. (2013). Inhibition of Botrytis cinerea spore germination and mycelia growth by frequency-specific sound. J. Korean Soc. Appl. Biol. Chem..

[79] Barreto J.A.R., Anaguano A.H. (2015). Evaluación del crecimiento y compatibilidad de hongos de la podredumbre blanca Evaluation of Growth and Compatibility of white Rot Fungi. Cienc. Desarro..

[80] Piermann L., Fujinawa M., Pontes N., Galvão J., Bettiol W. (2023). Inhibition of mycelial growth, conidial germination, and Botrytis cinerea Pers.: Fr colonization in begonia with biocompatible products. Sci. Agric..

