# Supplementary material for: The Effect of Broccoli Glucosinolates Hydrolysis Products on Botrytis cinerea: A Potential New Antifungal Agent
Source: Int J Mol Sci. 2024 Jul 20;25(14):7945. doi: 10.3390/ijms25147945 (PMC11277183; doi:10.3390/ijms25147945)
Supplement: Supplementary file 1 [file ijms-25-07945-s001.zip › ijms-3029597-supplementary.pdf]

SUPPLEMENTAL MATERIAL

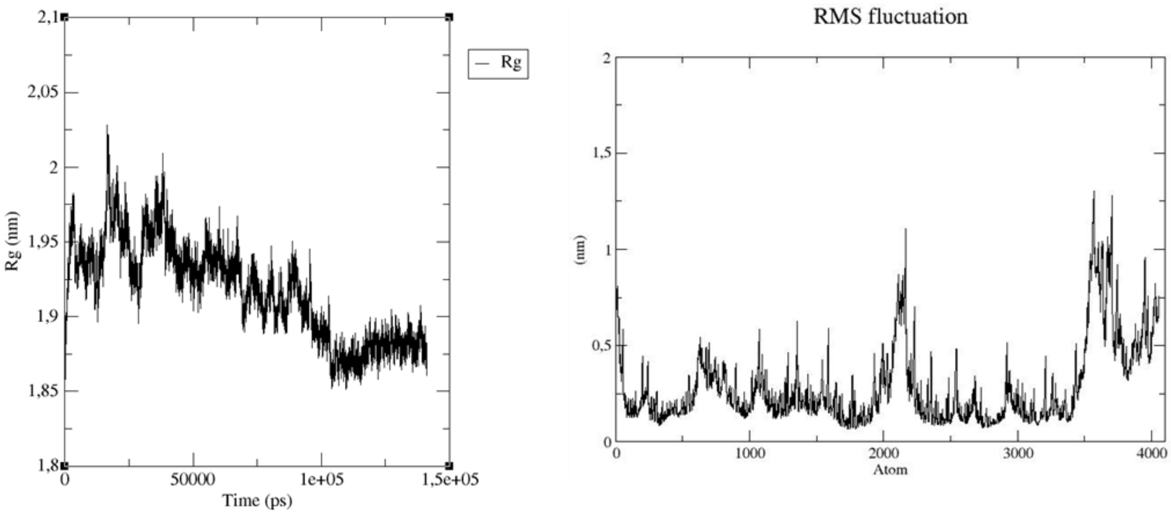

Figure S1. Radius of Gyration and RMSF Analysis for GST Enzyme

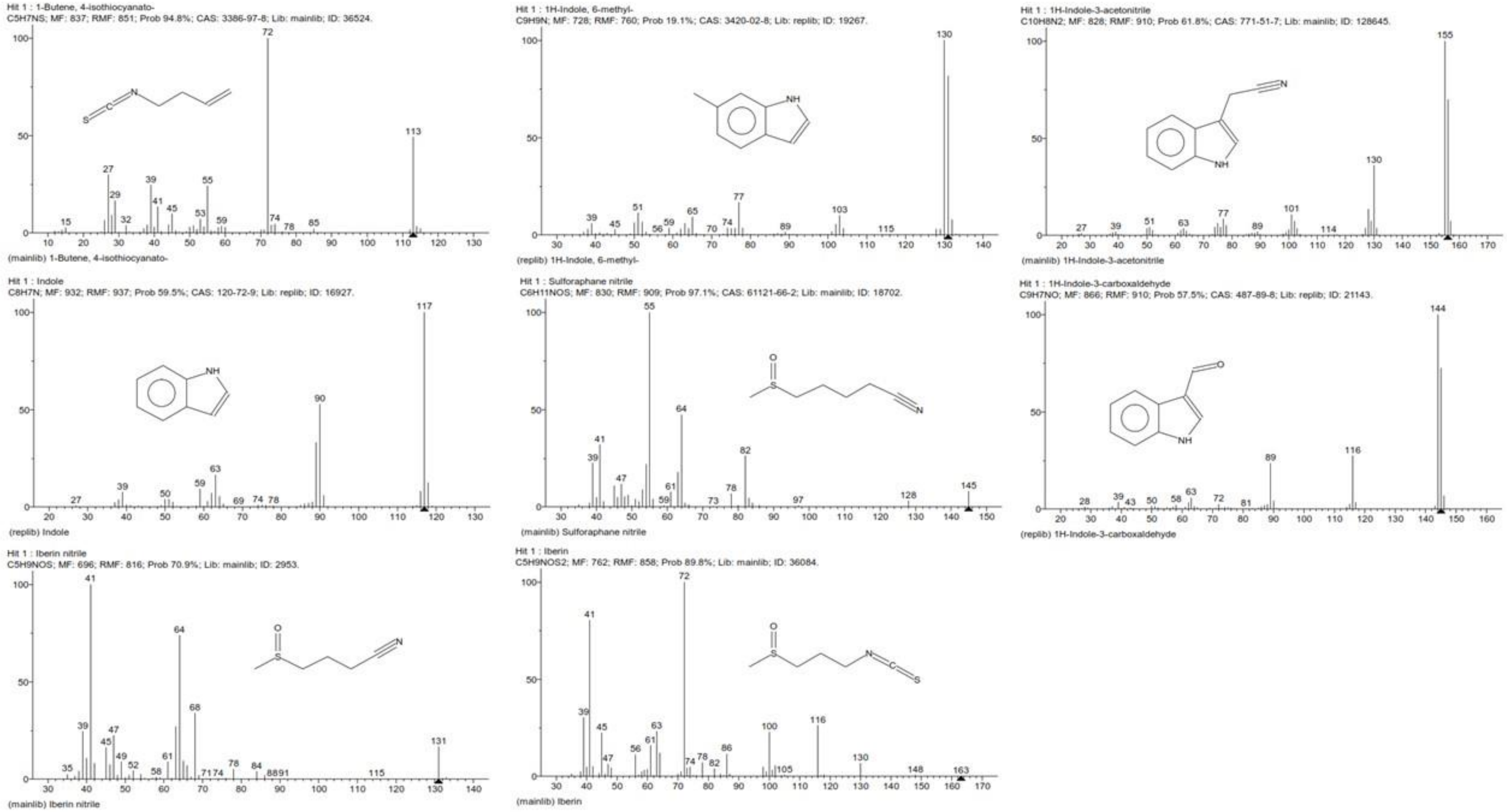

Figure S2. Mass Spectrometry Profiles of Glucosinolate Hydrolysis Products (GHPs) Identified in Broccoli Inflorescences, Stems, and Leaves. Each panel displays the mass spectrum for a different GHP, with key molecular ions and their corresponding mass-to-charge (m/z) ratios indicated.

Table S1. Comprehensive Validation Results of CYP51 and GST Models Using Various Structural Evaluation Tools.

Tool	CYP51 Results	GST Results
QMEAN	QMEANDisCo Global: 0.77 ± 0.05<br><br>(https://swissmodel.expasy.org/qmean/2j2tZH)<br><br>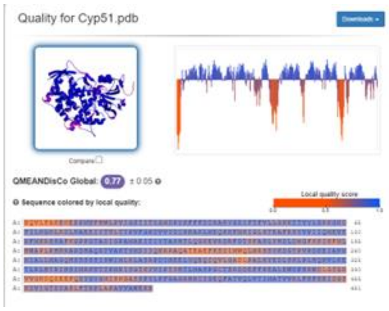	QMEANDisCo Global: 0.66 ± 0.05<br><br>(https://swissmodel.expasy.org/qmean/P336Cn)<br><br>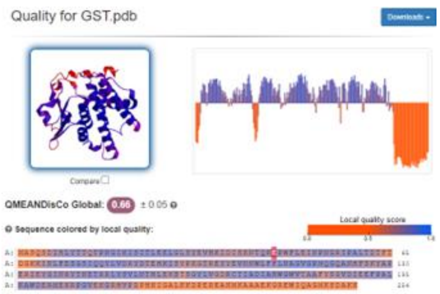
ERRAT	Overall Quality Factor: 94.2797<br><br>(https://saves.mbi.ucla.edu/results?job=1698296&p=errat)<br><br>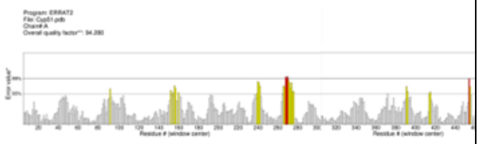	Overall Quality Factor: 82.9268<br><br>(https://saves.mbi.ucla.edu/results?job=1698299&p=errat)<br><br>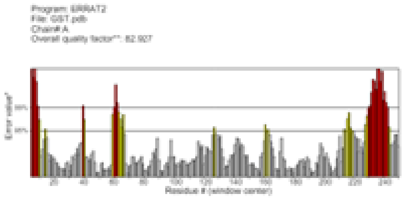

VERIFY3D	85.45% of residues scored >= 0.1 in 3D-1D profile<br>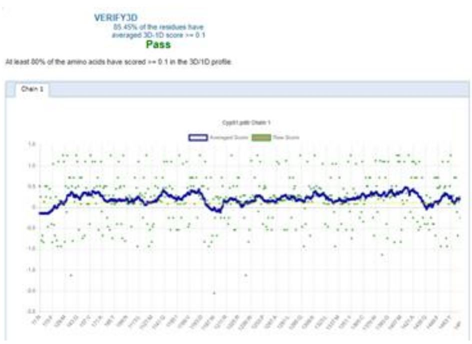 	91.34% of residues scored >= 0.1 in 3D-1D profile<br>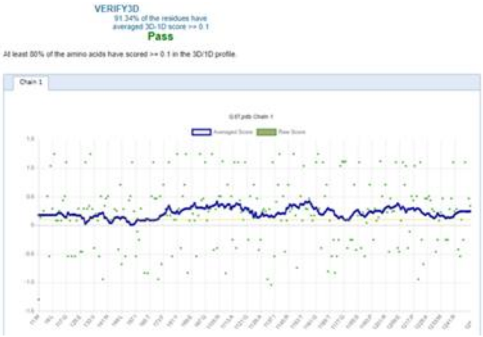
Tool	CYP51 Results	GST Results
PROCHECK	Ramachandran Plots:<br>6 errors, 1 warning, 1 pass<br>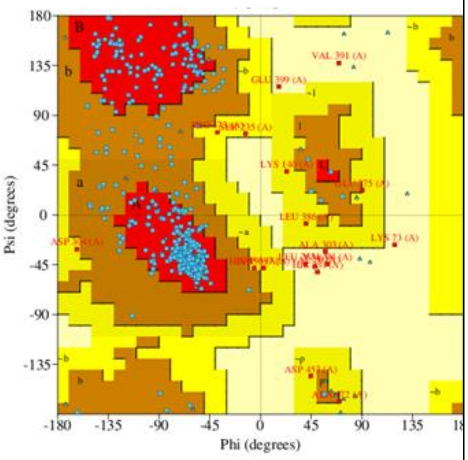 	Ramachandran Plots:<br>7 errors, 2 warnings, 0 pass<br>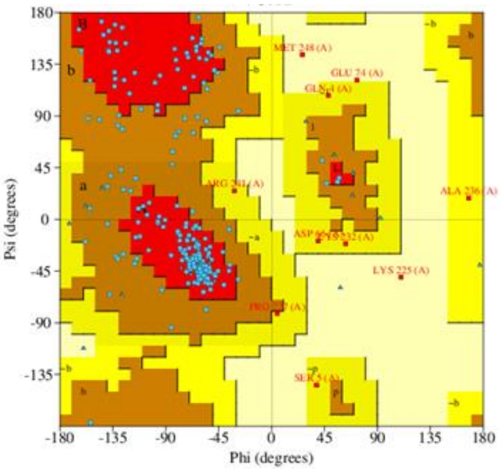

Tool	CYP51 Results	GST Results
ProSA	Z-Score: -9.27<br><br>(https://prosa.services.came.sbg.ac.at/prosa.php)<br><br>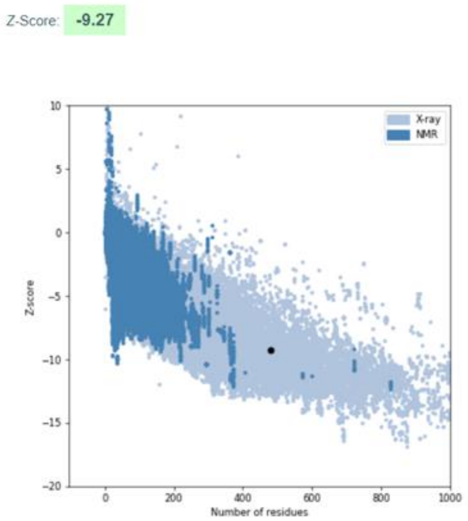	Z-Score: -4.08<br><br>(https://prosa.services.came.sbg.ac.at/prosa.php)<br><br>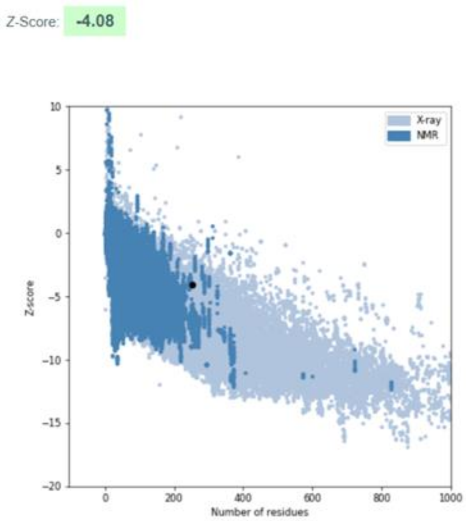
PDBSum	https://encr.pw/XjRDI<br><br>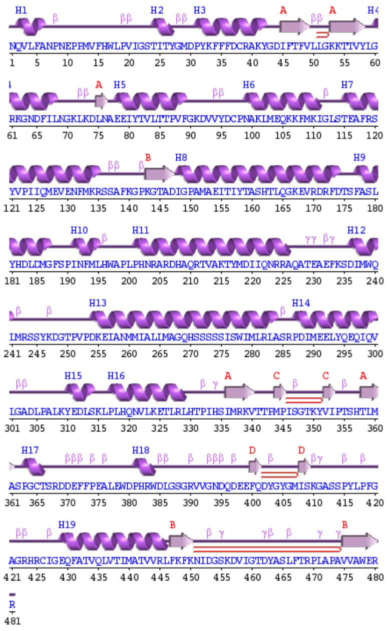	https://acesse.dev/d316d<br><br>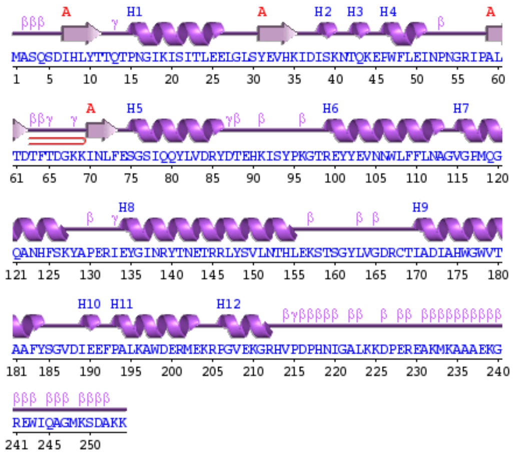
